# A Cost-Effective System for Aerial 3D Thermography of Buildings

**DOI:** 10.3390/jimaging6080076

**Published:** 2020-08-02

**Authors:** Claudia Daffara, Riccardo Muradore, Nicola Piccinelli, Nicola Gaburro, Tullio de Rubeis, Dario Ambrosini

**Affiliations:** 1 Dept. of Computer Science, University of Verona, str. Le Grazie 15, I-37134 Verona, Italy; claudia.daffara@univr.it (C.D.); riccardo.muradore@univr.it (R.M.); nicola.piccinelli@univr.it (N.P.); nicola.gaburro@univr.it (N.G.); 2 ISASI-CNR, Institute of Applied Science and Intelligent Systems, Via Campi Flegrei 34, I-80078 Pozzuoli (NA), Italy; 3 Dept. of Industrial and Information Engineering and Economics, University of L’Aquila, P.le Pontieri 1, I-67100 L’Aquila, Italy; tullio.derubeis@univaq.it

**Keywords:** infrared thermography, unmanned aerial vehicles, 3D modelling, energy efficiency, cultural heritage

## Abstract

Three-dimensional (3D) imaging and infrared (IR) thermography are powerful tools in many areas in engineering and sciences. Their joint use is of great interest in the buildings sector, allowing inspection and non-destructive testing of elements as well as an evaluation of the energy efficiency. When dealing with large and complex structures, as buildings (particularly historical) generally are, 3D thermography inspection is enhanced by Unmanned Aerial Vehicles (UAV—also known as drones). The aim of this paper is to propose a simple and cost-effective system for aerial 3D thermography of buildings. Special attention is thus payed to instrument and reconstruction software choice. After a very brief introduction to IR thermography for buildings and 3D thermography, the system is described. Some experimental results are given to validate the proposal.

## 1. Introduction

Three-dimensional (3D) imaging [1] is an important tool in many fields, ranging from industrial and architectural design to diagnostics of materials and artifacts, from medicine to entertainment (cinema, video games) and the fruition of historical and artistic heritage (augmented reality, virtual reconstruction). 

Infrared (IR) thermography (IRT) [2] is also a technique that has grown very rapidly in recent years, now characterized by increasingly advanced applications. Therefore, the joint use of these two techniques is of great interest and potential and represents a very current research topic.

3D thermography can be very useful, for example, in structural diagnostics, energy efficiency assessment of buildings, inspection and monitoring, and these evaluations may be enhanced by performing them from UAVs (Unmanned Aerial Vehicles—also known as drones) [3].

IRT is based on the fact that all bodies having temperature above absolute zero emit radiation; from this radiation, it is possible to trace the temperature of the body. Therefore, thermography is a method capable of detecting the temperature of objects under investigation without contact.

**Figure 1:**
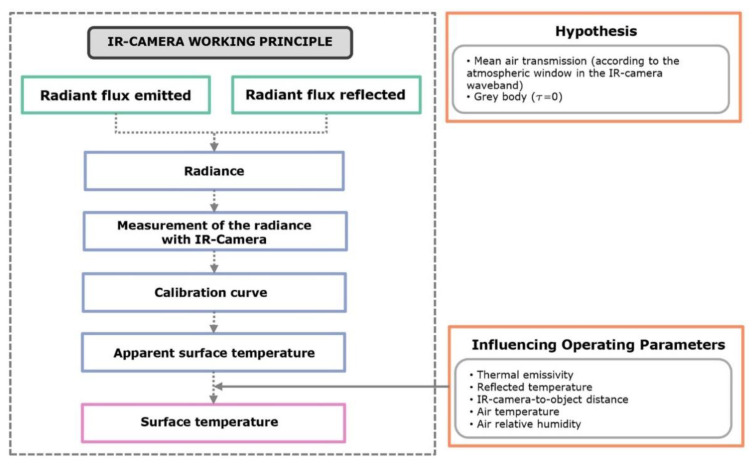
Basic principle of a thermal camera.

A thermographic camera is a calibrated device capable of measuring the radiation emitted by objects and calculating their temperature. The radiation measured by their sensor also depends on the properties of the investigated surface (emissivity) and the environment (radiation absorbed or emitted by the atmosphere between sensor and object and contribution of other objects in the environment). Basic principles are summarized in Figure 1.

The result is an image of the object in which the color or gray levels correspond to the different temperatures on the object’s surface. The measurement accuracy also depends on parameters such as ambient temperature, wind or solar radiation. Possible variations in temperature may be due to differences in materials surface finish (intrinsic or as a result of ageing/damage) or subsurface defects [2].

Diagnostic capabilities can be enhanced by a quantitative analysis of the thermographic data. Here, we are particularly interested in IRT for buildings, a flourishing application. An interrogation of the database SCOPUS using the search term “thermography AND buildings” in “article title, abstract and keywords” returned about 1725 papers, while the same interrogation only in “article title” returned 178 papers (data accessed 4 May 2020). Table 1 reports some recent references, identifying review papers and the main topics discussed.

The breadth of applications gives a fair idea of the current importance of IRT in buildings diagnostics and evaluations.

A further step ahead relates to 3D imaging. 3D imaging and displays are getting more and more important. For a general review of the topic, the interested reader is referred to References [1,30]: a comprehensive handbook and a recent extensive tutorial.

The great interest in 3D technologies also flourished in cultural heritage and buildings studies [31,32,33,34]. All this naturally leads to 3D thermography that is usually realized by combining 3D geometric data and two-dimensional (2D) thermographic data [35], and different setups are available according to the different 3D geometric acquisition systems and the different data fusion. Thus, for example, 3D geometry and 2D thermal images can be simply compared [36], infrared images can be mapped to 3D point clouds [37,38], integrated at different times in a Building Information Model (BIM) [39] or associated with a high-quality color laser scanner for cultural heritage monitoring and documentation [40].

The idea for the present work stems from the observation that several economical yet reasonably well-performing thermal cameras are on the market. They are integrated in smartphones (e.g., CAT S60 and CAT S61) or can be added to them as external modules (e.g., FLIR One and Seek Thermal) and available as stand-alone devices (e.g., FLIR C2). As their prices are typically within $1000, they help to increase the spread of IRT applications in many fields, such as, for example, biomedicine [41,42], agriculture [43], buildings inspection [44], cultural heritage diagnostics [45] and mass human temperature screening [46].

In this work, we propose a simple and cost-effective system to perform 3D aerial thermography of buildings. Particular attention is devoted to the choice of instruments and software for reconstruction. The article is structured in the following way: we describe the proposed system, with details on the choice of instruments, calibration and reconstruction software. The system was validated in a virtual environment. Finally, we show some experimental results.

## 2. Materials and Methods

### 2.1. A Cost-Effective System 

This paper is devoted to proposing a simple and cost-effective system for aerial 3D thermography of buildings.

To this aim, some key features of the system can be defined:

1. Thermal and geometric data should be recorded by the same device and in a single measurement process.
2. This device should be commercially available and cost-effective.
3. The reconstruction software should not require images taken by more than one recording device.
4. The reconstruction software should be as simple as possible without many parameters to tune.

### 2.2. Choice of Recording Device

**Figure 2:**
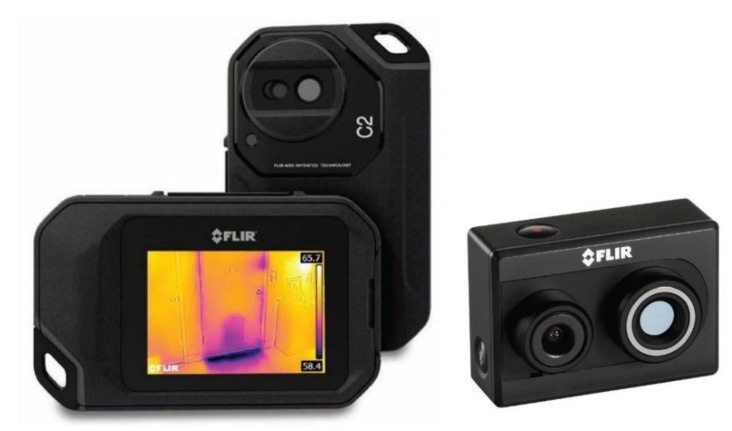
FLIR C2 (left) and FLIR Duo R (right) [47,48]. Pictures are not in scale.

Recent developments in sensor technology led to the production of miniaturized bolometers with LWIR (Long Wavelength Infrared band: 8–14 μm) sensitivity that are commercially available as camera core or mounted in compact thermal cameras. Two thermal cameras were chosen from the FLIR family of compact ones with visible light imaging: FLIR C2 and FLIR Duo R, and their respective features are summarized in Table 2. FLIR C2 was presented as the “world’s first full-featured, pocket-sized thermal camera designed for building industry experts and contractors” [47], while FLIR Duo R was presented as “the world’s first compact, lightweight, radiometric thermal and visible light imager designed for drone applications” [48]. Currently, the FLIR Duo R camera is no longer on the market and a new version is available (FLIR Duo Pro R). Figure 2 shows the two recording devices. The important feature is the simultaneous acquisition of a dual visible-thermal image dataset. The larger field of view and sensor size of the Duo R makes it more suitable for thermal acquisition of large-scale objects, and the higher resolution of the visible sensor allows for capturing a reliable dataset for 3D reconstruction.

### 2.3. Calibration Procedure of Visible-Thermal Sensors

The calibration boils down to the solution of a geometric problem: the estimation of the relative spatial position of the imaging sensors (visible and thermal). The key aspect here is that visible and thermal images are acquired in a single measurement process, therefore the calibration target must be suitable for the two imaging modalities, namely the reflective- and the emissive-based. A multi-step procedure is adopted only in the processing phase.

#### 2.3.1. The Calibration Passive Target

**Figure 3:**
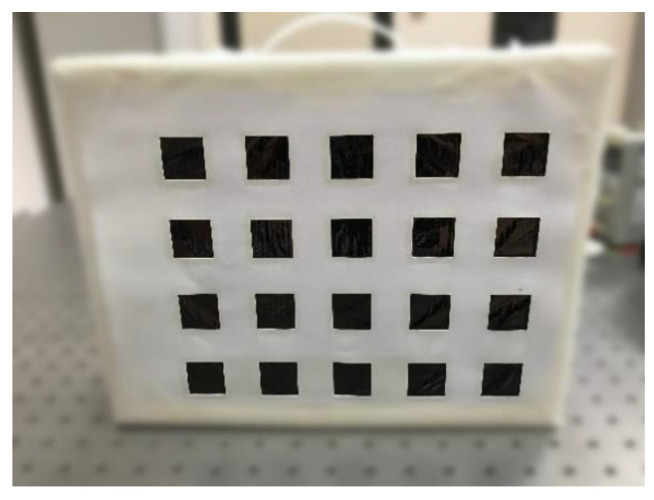
The calibration target.

Calibration targets can be of different types depending on (i) the markers used (corners, circles, etc.), (ii) their arrangement (structured or unstructured) and (iii) the working principle (active, passive) [49]. In this paper, a simple target (shown in Figure 3) was chosen to be cost-effective and easy to deploy. According to the previous taxonomy, it is based on squared features (grid pattern), and it is structured and passive (it does not require external energy sources, e.g., lightbulbs).

The target has been properly designed for the calibration of both the visible and the LWIR thermal sensor. A layer of material with a known emissivity value is covered by a second material, with cropped squares, having very different emissivity, giving rise to a geometric pattern, like a chessboard. The difference in emissivity in the two zones results in two different radiation values emitted, creating distinguishable zones in the thermal images. In particular, the masked layer is made by aluminum paper well laid and painted black (emissivity around 0.3), and the cover is white cardboard (emissivity around 0.9). The use of two separate physical layers, i.e., not of a painted pattern on single support, allowed limiting the blurring due to thermal diffusion at the interfaces, thus producing a sharper pattern. The problem of the specular reflection in the thermal range was addressed by applying a finishing (micro-roughness) to the aluminum to obtain a diffusive surface at LWIR wavelengths. This was simply done by pressing the foil on a sandpaper with a course grid size.

#### 2.3.2. The Calibration Algorithm

**Figure 4:**
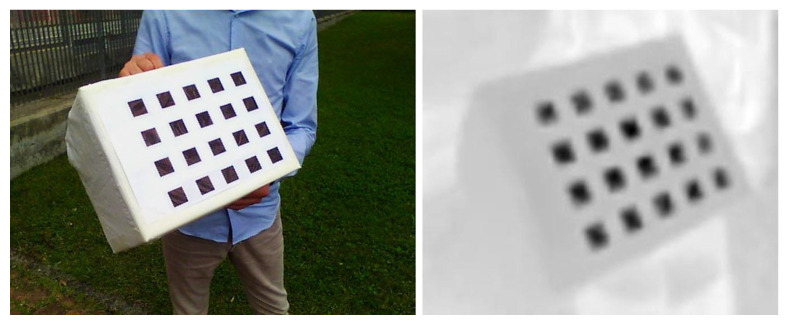
The target recorded in the visible (left) and in the thermal bands (right) in a single measurement.

The extraction of salient features from the visible images is straightforward, thanks to the high contrast and sharpness of the pattern and the imaging performance of CCD (Charge-Coupled Device) cameras. The identification of the internal regions was carried out by applying an intensity-based blob detection algorithm [50] to find the square centroid of each grid element. Clearly, besides the limited size of the bolometric sensor, the thermal images are less sharp than visible ones due to the nature of LWIR imaging itself. Thermal contrast is affected by the contribution of the environment that causes spurious reflections and makes feature extraction more difficult to achieve. Figure 4 shows an example of the target recorded at the same time in the two different bands.

Some specular contributions on the aluminum squares still occur in both the thermal and visible range and the problem was treated with a dedicated pipeline in the calibration phase, as detailed in the following.

**Figure 5:**
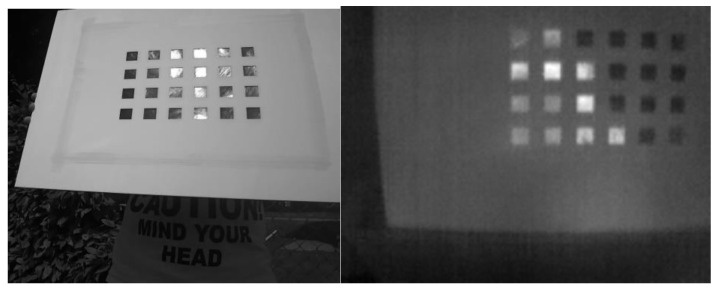
The reflections affecting the target, in the visible (left) and in the thermal bands (right).

First, the images are segmented using the Maximum Stable Extremal Region blob detector (MSER) [50]. For each image, the resulting blob set, which may contain outliers, is refined, imposing a set of shape constraints on each element. The resulting set is then dilated in order to isolate the single connected component containing the calibration pattern. Using the Hough transform, a set of lines is fitted along the edges of the connected component and the homography matrix is estimated. The image perspective is corrected by applying the homography transformation and, if the squares are not fully detected in the initial phase, an auxiliary search routine is applied around the first neighbors of the detected square. This routine applies a local clustering using an adaptive k-means and searches for corrupted squares. Assuming to have a good estimation of the square size, this auxiliary procedure clusters the grey level around each neighbor into higher and lower intensities with respect to the calibration mask grey level. These clusters are then merged, and the resulting region is checked against a set of shape constraints. If this new region satisfies the constraints, it is assumed to be a new square and added to the detected set. The procedure is iteratively applied until the detected set is full or the maximum iteration is reached. If the image is not rejected, a set of horizontal of vertical lines are fitted into the points and the set of square centroids is substituted with the corresponding line intersections. The last step is to apply the stereo intrinsic and extrinsic estimation proposed in Reference [51]. The proposed calibration procedure allowed to mitigate the possible reflections on the calibration target in the visible and thermal bands, which have their own characteristics (see Figure 5) and degrade the effectiveness of a classical geometric calibration at a different level. Combining state-of-the-art computer vision algorithms, it was possible to recover the corrupted square regions, reducing the number of the undetectable images. This allowed to reduce the calibration time and to perform the calibration in an uncontrolled environment with a passive target, without the need of heating sources.

The calibration method has been successfully applied both indoors and outdoors, obtaining comparable estimation error, and a relative rotation between the sensors of zero, as expected. The very small calibration error allows the 3D reconstruction to be effectively performed, as shown later.

### 2.4. Validation on Virtual Environment

**Figure 6:**
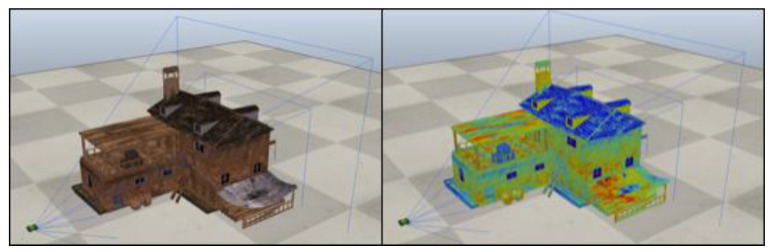
The virtual environment with the texture for the visible (left) and for the thermal bands (right).

To validate the reconstruction algorithm, a virtual environment was initially used. The simulator used during the testing for the aerial 3D reconstruction was *CoppeliaSim*, an open-source robotics simulator with interfaces to multiple programming languages. The simulation environment (shown in Figure 6) was composed of a teleoperated quadrotor, a multi-texturized model of a building, a model of a stereo vision system and a virtualized GPS.

**Figure 7:**
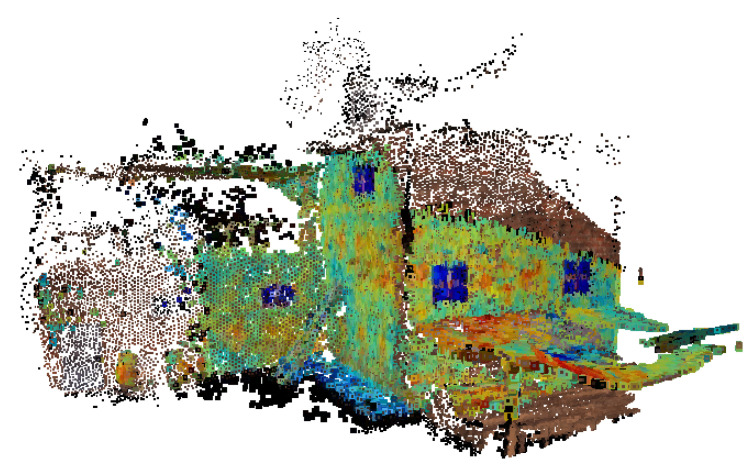
Superposition of the dual visible-thermal three-dimensional (3D) reconstruction of the virtual environment.

The vision system has been configured to mimic the behavior of a realistic thermographic camera setup, specifically by setting the nominal field of view, sensor size and frame rate of the FLIR Duo, together with the results from the geometric calibration for the relative positioning of the visible and thermal sensors. Finally, to simulate the appearance of the building in the reflective and emissive imaging modes, we applied two specific textures which are then “seen” either by the visible or by the thermal simulated sensor, respectively. The simulator has been interfaced to MATLAB, from where we computed and controlled the drone trajectory and the image acquisition. Figure 7 shows the 3D reconstruction using the images acquired by the simulated drone.

### 2.5. 3D Reconstruction Pipeline

The Structure from Motion (SFM) technique was chosen as the reconstruction algorithm. SFM [52,53,54,55,56,57,58,59] is a photogrammetry technique able to reconstruct a sparse 3D model of a static target from several 2D images of the same object taken from different points of view. To get a dense 3D reconstruction, the resulting sparse model must be further elaborated using a multi-view stereo (MVS) [59] algorithm. The SFM and MVS methods used for the reconstruction were provided by the open-source projects OpenMVG (Open Multiple View Geometry) [60] and CMVS (Clustering Views for Multi-View Stereo), respectively.

The proposed reconstruction methodology uses the visible images for building the full 3D object reconstruction by SFM and MVS. Then, thanks to the geometric calibration of the dual visible-thermal sensor, for each 3D point of the reconstructed model, the corresponding radiometric thermal value is computed. This is accomplished by keeping track, for each 3D point, of the pair of images from which it has been triangulated and then projecting it back into the image plane of one of them. Once in the image plane, its coordinates (in pixels) are transformed from the visible image frame into the related thermal frame (through the homogeneous transformation estimated with the geometric calibration presented in Section 2.3), where the radiometric value can be obtained. By performing this reprojection for each 3D point, it is possible to derive the final radiometric 3D thermal model. Clearly, due to the different sensor resolution, the thermal mapping is not bijective anymore. In our approach, the thermal images are not subjected to any super sampling or other interpolation technique, and multiple pixels of the visible images are simply mapped into a single thermal pixel and, consequently, to the same temperature. The advantage of this approach is that the temperatures mapped in the point cloud are the real values recorded by the thermal sensor.

In order to build the visible 3D model, the first step is to extract the conjugated features for each image. The discriminative capabilities of these features heavily affect the performance and the quality of the overall Structure from Motion. In our method, we adopted A-KAZE (Accelerated KAZE), a fast multi-scale feature detection and description method based on nonlinear scale spaces [61].

### 2.6. Mission Planning and Drone Control

The trajectory for the image acquisition is planned before the mission using *Pyfplanner,* an open-source software, developed by the authors in Python, and available online at https://gitlab.com/npiccinelli/pyfplanner. The aim of the software is to provide a sequence of commands to later be sent to the Unmanned Aerial Vehicle (UAV) through the open-source ground station software *Mission Planner* (https://ardupilot.org/planner/index.html). Table 3 lists the available commands.

The trajectory generated by the software is on the plane along the vertical direction of the line connecting the initial and the final position. The positions are defined in geographic coordinates. The maximum height of the trajectory is defined in meters with respect to a ground offset in order to avoid an undesired hovering effect. If the camera field of view (FOV) and the acquisition distance are known in advance, it is possible to derive the waypoint distance based on the desired overlap percentage between neighbor images. Otherwise, the software allows to set the vertical and horizontal traverse steps manually. In the case of a visible-thermal stereo system with different FOVs (e.g., such as the FLIR Duo R), to guarantee the full coverage by the two sensors, the FOV used to plan the trajectory should be the smaller one.

## 3. Results and Discussion

The goal of this work was to propose an effective and simple-to-use workflow for 3D thermography of buildings by exploiting dual visible-thermal sensors mounted on Unmanned Aerial Vehicles. The method has been validated using only the FLIR Duo R model because even if the FLIR C2 is a dual visible-thermal camera, the limited size of the visible sensor does not provide enough spatial resolution when applied in the field of aerial 3D reconstruction. In fact, to have a dense 3D reconstruction, imaging resolution must be high enough to capture the texture of the reconstructed surface. At a typical distance of 5 m from the building, the FLIR C2 cannot guarantee a good enough reconstruction; to be more specific, the size of the image cell at object plane, at a distance of 5 m, is about 8 mm for the FLIR C2 and 4 mm for the Duo R, corresponding to a minimum resolved detail of 16 and 8 mm respectively, according to Nyquist. Moreover, as the FLIR Duo R has been specifically designed to be carried around with a drone, it has a ready-to-use interface with the drone communication system MAVLink.

### 3.1. Experimental Setup

The proposed solution was experimentally validated in a noncontrolled environment by performing the outdoor 3D reconstruction of a building under restoration. The image acquisition was done near the city of Verona (Italy) in mid-April 2019, with cloudy conditions and atmospheric temperature between 9 and 13 °C. The UAV used was a custom-made quadrotor controlled through a Pixhawk running ArduPilot, an open-source project based on the Arduino framework. The quadrotor was also provided with an electronic gimbal to control the rotation of the vision system. The UAV flight plan was made using *Pyfplanner* and uploaded in the UAV controller using Mission Planner.

**Figure 8:**
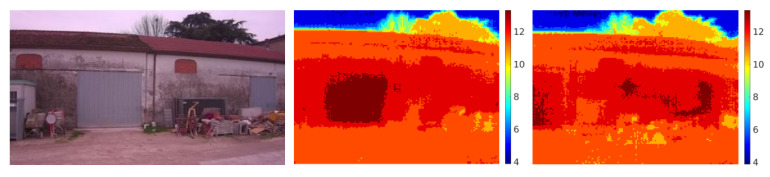
Images from the dataset. Picture of the building in the visible band (left) and same view captured in the thermal band (middle and right). The thermal range is between 3 and 14 °C.

The trajectory was designed to provide a sequence of vertical and horizontal images with 80% of overlap, a façade distance of 10 m, for safety reasons, and maximum height of 8 m. The acquired dataset is composed of 37 pairs of visible and thermal images (an example is shown in Figure 8). The whole measurement process took 5 min. The 3D reconstruction and the thermal mapping run on a i7 8700 with 32 GB of RAM and took about 15 min to accomplish the dense reconstruction.

### 3.2. 3D Reconstruction

**Figure 9:**
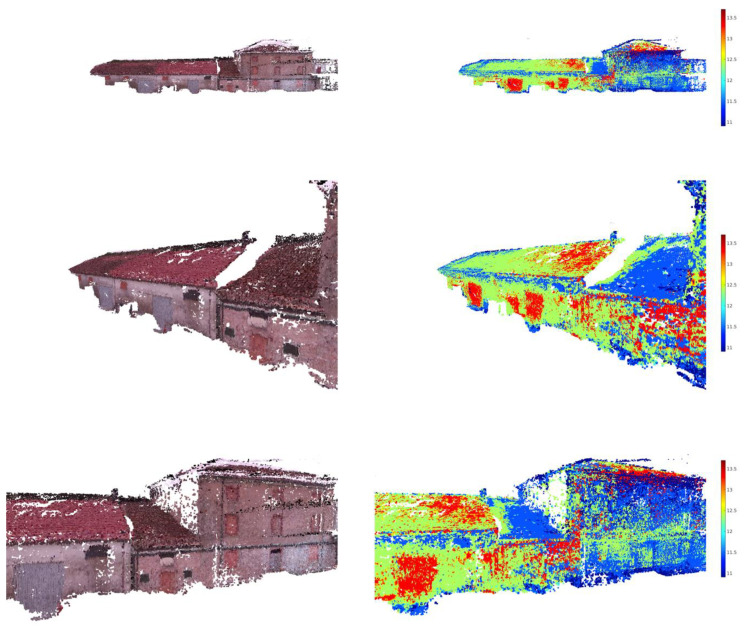
Different views of the building from the aerial 3D reconstruction in the visible band (left column) and thermal band (right column). The thermal range of the 3D reconstruction was adjusted between 11 and 13.5 °C.

The resulting 3D reconstruction is shown in Figure 9, and even without a quantitative evaluation of the mapping accuracy, it can be seen how the proposed method is able to map the thermal information over the 3D reconstruction with acceptable accuracy.

## 4. Conclusions

3D thermography can be an unparalleled tool for building diagnostics. When used by drones, it can allow safe inspection of parts that are difficult to reach or that would be difficult to examine in any other way (such as roofs). Applications can range from structural or maintenance diagnostics to the investigation of large archaeological sites and energy audits. 3D thermography, generally always associated with a 3D representation of the building in the visible band, has the key feature of allowing an accurate location of the thermal map.

In this article, we have proposed a simple system to realize an aerial 3D thermography of buildings. The system consists of a single device, which takes 2D images simultaneously in the visible and long infrared bands. After calibration, it is possible to reconstruct the 3D in the visible band with SFM techniques and then add the thermal information. The system has been validated during a real measurement campaign from a drone on a civil building. 

Laser scanning can provide a great amount of data, in the form of a point cloud dataset, but instrumentation is costly and requires a highly skilled operator. Also, LiDAR (Light detection and Ranging) instruments can provide 3D data with high spatial precision but once again, at a high cost. Although the obtained results cannot compete with those provided by these more sophisticated instrumentations, we also consider the performance of the proposed simple and cost-effective system very interesting in the continual monitoring of historical buildings and 3D objects, e.g., statues. 

## Figures and Tables

**Table 1:** A (not exhaustive) list of recent papers about infrared thermography (IRT) for buildings.

Authors	Year	Main Topic	Notes
Garrido et al. [4]	2020	Post-processing	Review
Huang et al. [5]	2020	Facades diagnostics	
Teni et al. [6]	2019	Thermal transmittance	Review
Bienvenido-Huertas et al. [7]	2019	Thermal transmittance	Review
Soares et al. [8]	2019	Thermal transmittance	Review
Glavaš et al. [9]	2019	Cultural heritage	
Royuela-del-Val et al. [10]	2019	Air infiltration	Neural network
Nardi et al. [11]	2018	Heat losses	Review
Kirimtat et al. [12]	2018	Thermal performance	Review
Baldinelli et al. [13]	2018	Thermal bridges	
Lucchi [14]	2018	Energy audit	Review
Lerma et al. [15]	2018	Air infiltration	
O’Grady et al. [16]	2017	Heat losses	
Barreira et al. [17]	2017	Air leakage	
Fox et al. [18]	2016	Diagnostics	
Nardi et al. [19]	2016	Thermal transmittance	
Djupkep Dizeu et al. [20]	2016	Indoor conditions	
Barreira et al. [21]	2016	Moisture	
Sfarra et al. [22]	2016	Cultural heritage	Solar heating
Fox et al. [23]	2015	Diagnostics	
Albatici et al. [24]	2015	Thermal transmittance	
Kylili et al. [25]	2014	Diagnostics	Review
Nardi et al. [26]	2014	Thermal transmittance	
Krankenhagen et al. [27]	2014	Cultural heritage	Solar heating
Paoletti et al. [28]	2013	Cultural heritage	
Dall’O’ et al. [29]	2013	Energy audit	

**Table 2:** Comparison of selected recording devices.

Imaging Specifications	FLIR C2	FLIR Duo R
IR sensor	80 × 60 pixels	160 × 120 pixels
Thermal sensitivity	<0.10 °C	<0.050 °C (*)
Field of view	41° × 31°	57° × 44°
Spectral range	7.5–14 μm	7.5–13.5 μm
Accuracy	±2 °C	±5 °C
Digital camera	640 × 480 pixels	1920 × 1080 pixels
Operating temp. range	−10 to +50 °C	0 to +50 °C
Weight (incl. battery)	0.13 kg	0.084 kg
Size	125 × 80 × 24 mm^3^	59 × 41 × 29.6 mm^3^

(*) nominal sensitivity of the Lepton core sensor https://www.flir.com/products/lepton/.

**Table 3:** Mission planning and Unmanned Aerial Vehicle (UAV) control command list.

UAV Commands	Parameters	Description
HOME	(latitude, longitude, altitude)	Define the home position for the UAV controller
WAYPOINT	(latitude, longitude, altitude)	Define a new waypoint to the current flight plan
CAMROT	(yaw, pitch, rool)	Set a camera rotation relative to the forward direction
SHOT	none	Trigger the shot command to the FLIR Duo R
CYAW	(yaw, pitch, rool)	Define the forward direction of the camera with respect to the initial rotation
LOITER	(latitude, longitude, altitude)	Keep the UAV in the commanded position
LAND	none	Start the landing procedure
